# The Role of Strigolactones and Their Potential Cross-talk under Hostile Ecological Conditions in Plants

**DOI:** 10.3389/fphys.2016.00691

**Published:** 2017-01-10

**Authors:** Sonal Mishra, Swati Upadhyay, Rakesh K. Shukla

**Affiliations:** Biotechnology Division, Central Institute of Medicinal and Aromatic Plants of Council, Scientific and Industrial Research (CSIR)Lucknow, India

**Keywords:** biotic stress, drought, nutrient starvation, salinity, strigolactone, temperature

## Abstract

The changing environment always questions the survival mechanism of life on earth. The plant being special in the sense of their sessile habit need to face many of these environmental fluctuations as they have a lesser escape option. To counter these adverse conditions, plants have developed efficient sensing, signaling, and response mechanism. Among them the role of phytohormones in the management of hostile ecological situations is remarkable. The strigolactone, a newly emerged plant hormone has been identified with many functions such as growth stimulant of parasitic plants, plant architecture determinant, arbuscular mycorrhiza symbiosis promoter, and also in many other developmental and environmental cues. Despite of their immense developmental potential, the strigolactone research in the last few years has also established their significance in adverse environmental condition. In the current review, its significance under drought, salinity, nutrient starvation, temperature, and pathogenic assail has been discussed. This review also opens the research prospects of strigolactone to better manage the crop loss under hostile ecological conditions.

## Introduction

The plant being sessile, need to manage their lifestyle under ongoing ecological constraints. In general, plant has the capability to sustain themselves under mild environmental changes. But in order to do so the plant makes changes in almost every aspect of their biology like morphology, anatomy, physiology, and molecular biology. It has been known since last few decades that the crop productivity is negatively influenced by biotic and abiotic stresses. Usually, crop responds by perception of these stress and subsequent signal transduction followed by the regulation at the molecular level. Among the array of candidates, phytohormones appeared to be a crucial tool to address the stress management. The plant hormones are key determinants of plant behavior, which, along with other signals like reactive oxygen species (ROS), Ca^++^, and other signaling molecules provide tools to acquire acclimatization under hostile environmental conditions. The plant hormones traditionally have been classified into growth promoting (auxin, gibberellins, cytokinins) or inhibiting (ethylene and abscisic acid) categories, thus particularly focused on development. The plant hormones have also been addressed for their role under stress conditions and the significance of phytohormones like abscisic acid (ABA), ethylene (ET), salicylic acid (SA), and jasmonic acid (JA) has been demonstrated.

In addition, the search of novel plant metabolites that may play role as phytohormones is one of the most fascinating researches for plant biologists. One such emerging candidate, strigolactones (SLs) are a small group of carotenoid derived compounds, exuded from the roots of 80% land plants and offer a symbiotic relation with soil arbuscular mycorrhiza (AM) (Akiyama and Hayashi, 2006). This new class of phytohormones control architecture of above and underground plant organ (Gomez-Roldan et al., 2008; Kapulnik et al., 2011; Kohlen et al., 2011; Ruyter-Spira et al., 2011). Beside this SLs also induce seed germination in root parasitic plants of the genera like *Striga, Orobanche, Alectra*, and *Phelipanche* (Yoneyama et al., 2013). These parasitic plants are usually incapable of photosynthetic assimilations thus depend on host for these requirements. Besides their impact on natural vegetation, their activity in agricultural land causes a serious problem for huge agriculture losses (Parker, 2009).

This hormone class got their name after the identification of its first candidate from *Striga* (Cook et al., 1966), hence strigolactone. Later studies demonstrated that this plant hormone suppresses shoot branching as the SL-deficient mutants were recognized, highly branched and the application of GR24 (a synthetic SL), may inhibit axillary bud development (Gomez-Roldan et al., 2008; Umehara et al., 2008). Thus, the prime investigation of SL has put views regarding its role as a growth/germination stimulant or symbiotic promoter. However, another important role of SL as a possible tool for stress related events have been described and its role is being documented in certain biotic and abiotic stress. In the current review, we have dealt with the ongoing progress of SL for maintenance of plant life under hostile ecological conditions. Such comprehensive compilation is important in present research era where the multilevel improvement approaches are preferred over conventional methods of crop improvement.

## Strigolactones—biosynthesis and importance

The first natural SL, strigol was discovered as a germination stimulant of *Striga lutea*, since then these compounds were collectively called as strigolactones (Cook et al., 1972). As root parasitic plants depend entirely on a host plant for water, assimilates, and nutrients so these plants not only devastate natural vegetation, but also are a major threat to commercial crops including maize, millet, sorghum, legumes, rapeseed, and tomato. In symbiosis between plant and AM fungi, SLs stimulates branching of fungal hyphae and help plant in obtaining available mineral nutrients particularly nutrient with low mobility such as phosphate.

It is reported that SLs derive from carotenoids as evident in maize plants with low SL accumulation after treatment with carotenoid biosynthesis inhibitor fluridone (Matusova et al., 2005). Functional role of SL can be correlated with its origin and biosynthesis as per the requirement of the system during evolution. As the gene involved in SL biosynthesis is found to be reported from different plant species including algae and bryophytes, so it can be hypothesized that these SLs are important molecule which has been sustained in the evolutionary chain from the long period. Basically SLs are four-ringed (A–D) compound, which shows the variation in their function due to attachment of different groups on A and B rings (Akiyama et al., 2010; Boyer et al., 2012). Initially SLs was considered as a sesquiterpene lactone, but later on it was revealed that they are apocarotenoid, which are actually derived from carotenoid cleavage mediated by Carotenoid cleavage dioxygenase (CCDs) enzymes (Booker et al., 2005). The member of CCD family is involved in the synthesis of different apocarotenoid like cyclohexenone and mycorradicin (Auldridge et al., 2006). The initial biosynthesis occurs in the plastids with the help of three plastid localized enzyme D27, CCD7, and CCD8.

**Figure 1:**
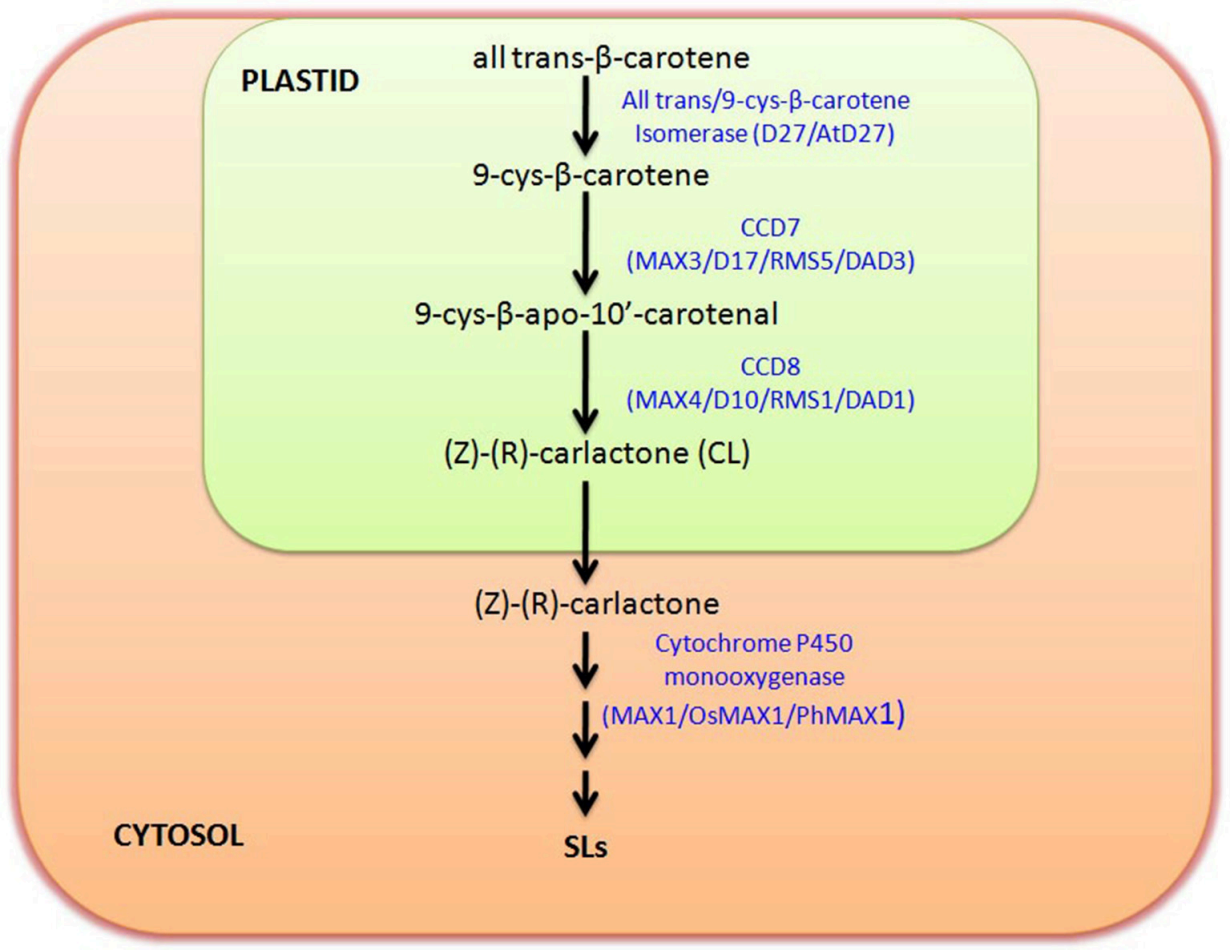
**Strigolactone (SL) Biosynthetic pathway:** figure shows the biosynthetic pathway of SL production and key enzymes involved in biosynthesis. SL biosynthesis occurs in two separate compartments, plastid and cytosol. Biosynthesis starts in plastid with the conversion of trans-β-carotene to (Z)-(R)-carlactone (CL) involving three intermediate steps catalyzed by trans/9-cys-β-carotene Isomerase, CCD7, and CCD8, respectively. Carlactone further moves to cytosol and there with the help of Cytochrome P450 monooxygenase (MAX1) and several other unidentified enzymes it is further converted to different other SLs. Enzymes involved in this biosynthetic pathway in different plants are named accordingly like Arabidopsis (MAX), Rice (D), and in Pea (RMS).

The genetic studies utilizing SL-deficient and SL-insensitive mutants have uncovered the SL biosynthesis and perception (Seto and Yamaguchi, 2014). Most of these mutants are shoot-branching mutants, like *ramosus* (*rms*) of *Pisum sativum, more axillary growth* (*max*) of *Arabidopsis thaliana, decreased apical dominance* of *Petunia hybrida* and *dwarf* or *high-tillering dwarf* (*htd*) of *Oryza sativa* (Beveridge et al., 2000; Booker et al., 2005; Zou et al., 2006; Simons et al., 2007). The SLs are derived from carlactone (CL), that itself synthesized as a result of sequential reactions by three biosynthetic enzymes, D27, CCD7, and CCD8 utilizing all-trans-β-carotene in plastids (Alder et al., 2012). This work has also demonstrated that CCD7 (carotenoid cleavage dioxygenase 7) cleaves 9-cis-β-carotene to produce 9-cis-β-apo-10′-carotenal, which is further utilized by CCD8 to give CL, a biosynthetic precursor for SLs. The CL is then further oxidized by cytochrome P450 monooxygenase MAX1 or other homologous genes into different forms of SLs by few other unidentified steps catalyzed by novel unidentified enzymes. The pathway of SL biosynthesis is shown in Figure 1. It has been reported that *max1* mutant accumulate high levels of CL (biologically inactive precursor), but still exhibited branching phenotype as that of *max3* and *max4* mutant plants, which was restored after SL treatment (Seto et al., 2014). With regard to perception of SLs, the participation of MAX2/D3/RMS4 (F-box protein) and D14/AtD14/DAD2 (α/77β-hydrolase) were noticed (Pandey et al., 2016).

The SLs are also involved in many aspects of plant development like coordination of growth and architecture of plants according to the availability of nutrients in soil. SLs regulates root growth and root hair elongation while on the other hand, they suppress secondary branching of the shoot (Koltai, 2011). SLs also stimulate secondary growth of stem and internode length in a cross talk with auxin and also regulate leaf senescence (Agusti et al., 2011; de Saint Germain et al., 2013; Yamada et al., 2014). The SL triggered positive regulation of *Sinorhizobium meliloti* induced nodulation was also observed in *Medicago sativa* (Soto et al., 2010). In Arabidopsis the SLs are involved in seed germination and seedling growth. It is reported that the ABA is involved in regulation of SLs biosynthesis, as in tomato there is downregulation of LeCCD7 and LeCCD8 genes responsible for SLs biosynthesis in ABA mutants *notabilis, sitiens*, and *flacca* (López-Ráez et al., 2010). Additionally, SLs behaves antagonistically to CK in bud outgrowth control (Dun et al., 2012). The positive regulation of drought and high salinity responses in Arabidopsis was observed by SLs and this feature was associated with shoot- rather than root-related traits. Moreover, in the process of stress management, plant utilizes integrative multiple hormone pathway that includes SL, ABA, and CK pathways; as evident from comparative transcriptome analysis (Ha et al., 2014).

The findings of recent research have answered many aspects of SLs particularly related with its biosynthesis, signaling, regulation of morphological/stress responses, and associated cross talk with different plant hormones during these events. In view of such diverse area of studies with regard to this novel plant hormone, the present study will uncover the role of SL under hostile environmental conditions.

## Strigolactones and hostile ecological conditions

**Figure 2:**
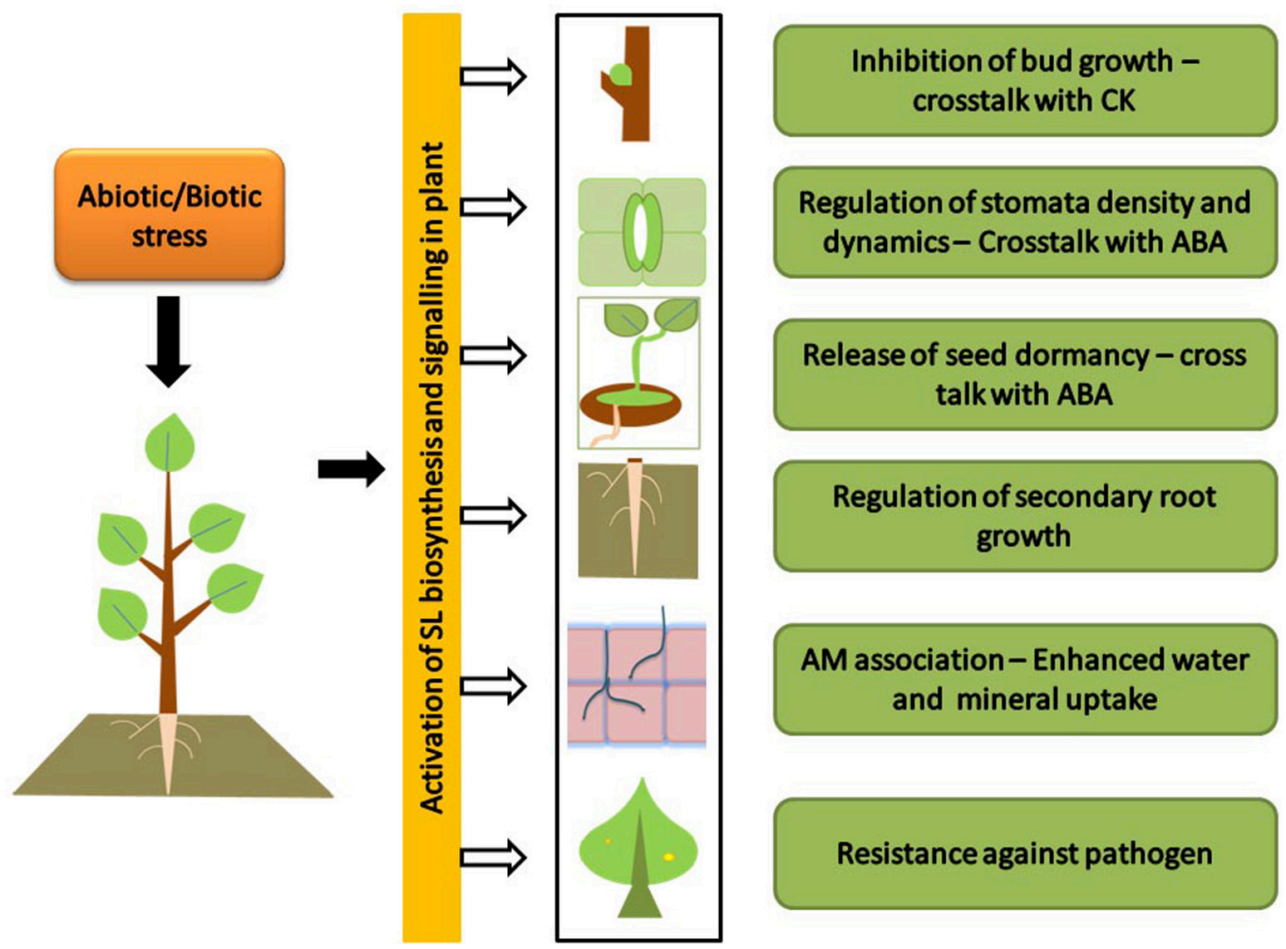
**Strigolactone triggered response under hostile ecological conditions:** picture shows the stress triggered biosynthesis and signaling of SLs. SLs under abiotic and biotic stress conditions provide resistance to plants. Under water stress SLs inhibit shoot growth (a cross talk with cytokinin) while enhance lateral root growth to increase the uptake of water. In a cross talk with ABA, SLs regulate stomatal density and release seed dormancy. SLs enhance arbuscular mycorrhizal (AM) association to increase mineral uptake under Nitrogen and Phosphorus deficiency. Under biotic stress condition SLs provide tolerance against pathogen infection.

The role of SL in stress and development has been dealt in a recent review (Pandey et al., 2016). Like other plant hormones, the prime function of SLs is the development and its interaction with auxin dominates in SL regulated developmental processes (Hayward et al., 2009). However, the recent exploration has also suggested the role of ABA in regulation of strigolactone production (López-Ráez et al., 2010), and thus possibility in stress management. Moreover, it has been noticed that the biosynthesis of SL are under the influence of nutrient starvation and regulation of SL production is a crucial response under stress conditions. In this context, the upcoming text will uncover the relation between strigolactone biosynthesis and its influence under hostile ecological conditions (Figure 2).

### Salinity and drought stress

The AM symbiosis alleviates the negative effects of salt stress on lettuce plants by shifting hormonal profiles. Aroca et al. (2013) have suggested that salt stress induced strigolactone production in AM associated lettuce plants further triggers AM fungal growth and thus helps to overcome stress conditions. In *Arabidopsis*, the positive regulation of drought and salinity response was observed through mediation of SL (Ha et al., 2014). The Arabidopsis *max3* and *max*4 mutants are sensitive to drought and salt stress and display higher stomatal density and delayed ABA-induced stomatal closure compared to wild type plants.

The role of SL in water stress has been observed, which was found to exhibit interplay with other plant hormones viz. ABA, etc. In one study, it has been demonstrated that the 9-*cis*-epoxycarotenoid dioxygenase (NCED)-inhibitor abamine and SG treated ABA deficient mutants' exhibit reduced ABA and SL levels. Moreover, the ABA mutants further revealed suppression of LeCCD7 and LeCCD8 transcripts (López-Ráez et al., 2010). It has been observed that the SL-deficient and SL-response mutants exhibited hypersensitivity to drought and salt stress and the features was associated with shoot related traits. Further studies with SL biosynthesis (max3 and max4) and response (max2) mutants exhibit that the SL treatment can rescue the germination under drought condition only in biosynthesis mutants. Additionally, these mutants also exhibit low sensitivity for ABA as compared to wild type plants (Ha et al., 2014). The comparison of *Lotus japonicus* wild-type and SL-depleted plants were studied under osmotically stressed and/or phosphate starved conditions. The stomatal conductance of SL-depleted plants was found higher and they exhibited weaker resistance in terms of slower stomatal closure in response to ABA, associated with drought (Liu et al., 2015). The SL pretreatment inhibited the osmotic-stress induced ABA production by suppression of *LjNCED2*. Recently, the *MAX2* identified from a parasitic plant (*Orobanche aegyptiaca, OaMAX2*) has also been characterized in Arabidopsis using complementary approach. Interestingly, *OaMAX2* restored the drought tolerant phenotype of *Atmax2* mutant, which suggested the conservation of *MAX2* signaling in parasitic and non-parasitic plants (Li et al., 2016). It is known that AM symbiosis alleviates drought stress in plants. Moreover, it has been noticed that the drought may influence the hormone profile in tomato and lettuce plants. Under such conditions, there is a possibility of high accumulation of strigolactone that in turn influences symbiosis thus is helpful to cope up with this stress (Ruiz-Lozano et al., 2016).

### Temperature

The temperature is one of the important environmental concerns that have been emerged mostly due to the recent way of developments. The plant requires a set of optimal conditions, including temperature, to perform their biochemical and physiological behavior and any short or long term fluctuations may bring them under stress. The seed germination in plants is dependent upon temperature and usually inhibited by high temperature. Again, plant hormones such as CK and GA positively influence germination processes and ABA negatively regulate the germination. SLs has been originally discovered as a chemical that stimulate the germination of seeds of parasitic weeds. However, it has been also noticed that SL also exhibit this activity for other plants. It has been observed that SL lowers the ABA to GA balance and further increases the cytokinin (CK) levels, which is a positive move toward seed germination. On application of GR24 under high temperature, the germination of SL defective *Arabidopsis* mutant gets stimulated (Tsuchiya et al., 2010). During warm stratification, the germination in dormant *Philipanche ramose* seed is rescued by SL that also involves a reduction of ABA levels (Lechat et al., 2015).

### Nutrient stress conditions

Under nutrient deprived conditions, plant produces high amounts of SLs that lead to suppression of shoot branching and stimulates symbiosis (Gomez-Roldan et al., 2008; Umehara et al., 2008). With their activity via modification of root and shoot architecture and promotional activity toward a symbiosis of rhizobial bacteria and AM fungi, SLs play crucial role in nitrogen and phosphorous deficiency (Marzec, 2016). In AM symbiotic relation, fungi ensure the supply of water and nutrients (particularly phosphate and nitrogen) through their hyphal extensions. Under low phosphate, the GR24 treated wild plants have been shown to demonstrate high lateral root numbers; however, the SL mutants exhibited low lateral roots. In contrast, the application of GR24 under high phosphate causes decrease in lateral root density by suppressing both outgrowth and lateral root-forming potential (Ruyter-Spira et al., 2011). This study advocated the participation of SL under these conditions. Further, the correlation of low phosphate, high strigolactones and reduced bud outgrowth was evident in *Arabidopsis* and rice (Umehara et al., 2010; Kohlen et al., 2011). Apart from the role of SLs in the management of phosphate homeostasis, the SLs have also been recognized as a possible route for regulating plant growth response to nitrogen supply. In a study with *Arabidopsis* mutants of SL-biosynthesis (*max1-1*) and SL-insensitive (*atd14-1*), the altered response to nitrogen deficiency was observed. Moreover, under limited nitrogen conditions the alteration of expression levels of SL biosynthesis genes (MAX3 and MAX4) was noticed (Ito et al., 2016).

### Biotic stress

Besides a role in growth and development, SLs has also been recognized as a player to offer resistance to specific pathogens (Marzec, 2016). First such evidence came with the identification of pathogen associated TF motifs in the promoter of genes associated with the SL biosynthesis (Torres-Vera et al., 2014). The study with SL synthesis and signaling impairs mutants of *A. thaliana* demonstrated the role of strigolactone in plant resistance against infection of bacteria like *Rhodococcus fascians, Pectobacterium carotovorum*, and *Pseudomonas syringae* (Piisilä et al., 2015; Stes et al., 2015). The infection of *R. fascians* resulted into more pronounced leafy gall syndrome in SL-biosynthesis (*max1, max3*, and *max4*) and signaling (*max2*) mutants. The supplementation of GR24 (a synthetic analog of SL) and D2 (an inhibitor of SL biosynthesis) in the media of wild types displayed greater susceptibility to *R. fascians*, only in later study. Moreover, the infection also triggers up regulation of genes associated with SL production (*max1, max3*, and *max4*; Stes et al., 2015). The radial growth of diverse phyto-pathogenic fungi was inhibited after application of GR24 (Dor et al., 2011). The increase in sensitivity toward *Alternaria alternata* and *Botrytis cinerea* was also observed in the *Lycopersicon esculentum* SL-biosynthesis mutant *slccd8* (ortholog of the *A. thaliana MAX4*), which was accompanied by reduction in the content of plant defense hormones (Torres-Vera et al., 2014). Contrary to this, SLs was not displayed their role in resistance to other pathogens viz, *Pythium irregulare* and *Fusarium oxysporum* (Blake et al., 2016; Foo et al., 2016). Thus, it can be stated that the SL may be involved in plant immune response involving only specific bacterial and fungal pathogens.

## Conclusion and future prospective

The interaction of phytohormones and stress is well known events in plant stress management. The current list of these candidates involves both the classical and newly discovered phytohormones and the list is still continuing with the advent of novel growth stimulatory phytochemicals. The plant hormones in most of the cases are originally defined and described in terms of development and its role in stress is usually being given the next preference. But, the recent outlook for key hormones like ABA, ET, JA, SA has put the clear categorization of plant hormones under two separate heads i.e., development and stress related. In this continuation, the SL has been recognized as an important emerging player in plant stress management.

The SL was originally described to favor AM symbiosis establishment and offer as host detection cues for root parasitic plants such as *Striga* and *Orobanche* and stimulate seed germination of these parasitic weeds (Bouwmeester et al., 2007; López-Ráez et al., 2011). The stress may influence directly the SL biosynthesis or its signaling or its cross-talk with other plant hormones particularly ABA. Mostly unique to this, they pave the way for plant survival growing under nutritional starvation by stimulating growth-supporting microbial community like AM and nodulation efficiency of nitrogen fixing bacteria. Furthermore, the impairment of either its synthesis or signaling may compromise pathogen specific plant resistance.

Though the significance of SL has been recognized in salinity, drought, temperature, nutrient starvation, and plant resistance, but its establishment as stress related hormones demand further studies. The queries that need to answer at this juncture may include—the disparity of SL biosynthesis/signaling in stress related contrasting genotype, their significance in multiple stress management to address the exact field problems, possibility of cross talk with other stress related hormones, interaction with plant transcription factors and related signaling pathway.

## Author contributions

SM has compiled the literature and written the manuscript. SU has helped in compilation of manuscript. All three authors have edited and revised the approved manuscript submitted.

## Funding

CSIR-NETWORK PROJECT BSC107.

### Conflict of interest statement

The authors declare that the research was conducted in the absence of any commercial or financial relationships that could be construed as a potential conflict of interest.
